# The IL-4/STAT6 signaling axis establishes a conserved microRNA signature in human and mouse macrophages regulating cell survival via miR-342-3p

**DOI:** 10.1186/s13073-016-0315-y

**Published:** 2016-05-31

**Authors:** Zsolt Czimmerer, Tamas Varga, Mate Kiss, Cesaré Ovando Vázquez, Quang Minh Doan-Xuan, Dominik Rückerl, Sudhir Gopal Tattikota, Xin Yan, Zsuzsanna S. Nagy, Bence Daniel, Szilard Poliska, Attila Horvath, Gergely Nagy, Eva Varallyay, Matthew N. Poy, Judith E. Allen, Zsolt Bacso, Cei Abreu-Goodger, Laszlo Nagy

**Affiliations:** Department of Biochemistry and Molecular Biology, Research Center for Molecular Medicine, University of Debrecen Medical, Nagyerdei krt. 98, H-4032 Debrecen, Hungary; Laboratorio Nacional de Genómica para la Biodiversidad (Langebio), Centro de Investigación y de Estudios Avanzados del IPN, Irapuato, Guanajuato 36821 México; Department of Biophysics and Cell Biology, University of Debrecen, Egyetem tér 1, H-4012 Debrecen, Hungary; University of Manchester, AV Hill Building, Oxford Road, Manchester, M13 9PT UK; Max Delbrueck Center for Molecular Medicine, Robert Roessle Strasse 10, Berlin, 13125 Germany; Sanford-Burnham-Prebys Medical Discovery Institute, 6400 Sanger Road, Orlando, FL 32827 USA; Genomic Medicine and Bioinformatic Core Facility, Department of Biochemistry and Molecular Biology, University of Debrecen, Nagyerdei krt. 98, H-4032 Debrecen, Hungary; National Agricultural Research and Innovation Centre, Agricultural Biotechnology Institute, Szent-Györgyi A. út 4, H-2100 Gödöllő, Hungary; MTA-DE “Lendület” Immunogenomics Research Group, University of Debrecen, Egyetem tér 1, H-4012 Debrecen, Hungary

## Abstract

**Background:**

IL-4-driven alternative macrophage activation and proliferation are characteristic features of both antihelminthic immune responses and wound healing in contrast to classical macrophage activation, which primarily occurs during inflammatory responses. The signaling pathways defining the genome-wide microRNA expression profile as well as the cellular functions controlled by microRNAs during alternative macrophage activation are largely unknown. Hence, in the current work we examined the regulation and function of IL-4-regulated microRNAs in human and mouse alternative macrophage activation.

**Methods:**

We utilized microarray-based microRNA profiling to detect the dynamic expression changes during human monocyte–macrophage differentiation and IL-4-mediated alternative macrophage activation. The expression changes and upstream regulatory pathways of selected microRNAs were further investigated in human and mouse in vitro and in vivo models of alternative macrophage activation by integrating small RNA-seq, ChIP-seq, ChIP-quantitative PCR, and gene expression data. MicroRNA-controlled gene networks and corresponding functions were identified using a combination of transcriptomic, bioinformatic, and functional approaches.

**Results:**

The IL-4-controlled microRNA expression pattern was identified in models of human and mouse alternative macrophage activation. IL-4-dependent induction of miR-342-3p and repression of miR-99b along with miR-125a-5p occurred in both human and murine macrophages in vitro. In addition, a similar expression pattern was observed in peritoneal macrophages of *Brugia malayi* nematode-implanted mice in vivo. By using IL4Rα- and STAT6-deficient macrophages, we were able to show that IL-4-dependent regulation of miR-342-3p, miR-99b, and miR-125a-5p is mediated by the IL-4Rα–STAT6 signaling pathway. The combination of gene expression studies and chromatin immunoprecipitation experiments demonstrated that both miR-342-3p and its host gene, EVL, are coregulated directly by STAT6. Finally, we found that miR-342-3p is capable of controlling macrophage survival through targeting an anti-apoptotic gene network including Bcl2l1.

**Conclusions:**

Our findings identify a conserved IL-4/STAT6-regulated microRNA signature in alternatively activated human and mouse macrophages. Moreover, our study indicates that miR-342-3p likely plays a pro-apoptotic role in such cells, thereby providing a negative feedback arm to IL-4-dependent macrophage proliferation.

**Electronic supplementary material:**

The online version of this article (doi:10.1186/s13073-016-0315-y) contains supplementary material, which is available to authorized users.

## Background

Macrophages display substantial functional heterogeneity, allowing them to participate in diverse aspects of the immune response, including immediate defense against pathogens, regulation of lymphocyte activation, and clearance of cell debris and microbes through phagocytosis, and to contribute to tissue regeneration [1, 2]. Macrophage polarization states and functional properties are determined by the tissue microenvironment containing cytokines, different pathogen-derived molecules, as well as lipid mediators [3]. Two well-established end points of macrophage polarization are classic (M1) and alternative (M2) macrophage activation induced by the Th1-type cytokine interferon gamma and bacterial lipopolysaccharide (LPS) and the Th2-type cytokines interleukin (IL)-4 and IL-13, respectively [3, 4]. M1-type macrophage activation is triggered either by the activation of the Janus kinase (JAK)/signal transducer and activator of transcription (STAT1) axis or the activator protein 1 (AP-1) and nuclear factor kappa-light-chain-enhancer of activated B cells (NFkB) signaling pathways, resulting in enhanced bactericidal capacity and pro-inflammatory properties [3, 5]. In contrast, IL-4 activates the IL4Rα/JAK/STAT6 and phosphoinositide 3-kinase (PI3K) pathways, both of which contribute to alternative macrophage activation [6]. M2-type macrophages possess a characteristic gene expression signature endowing them with anti-inflammatory and immune regulatory properties. The significance of alternative macrophage activation has been described in a range of physiological and pathological processes, including hypersensitivity, anti-helminthic immune responses, fibrosis, sepsis, and tumor progression [4, 6–8]. The functional properties associated with distinct macrophage activation states require tight but plastic regulation of activation-specific gene expression programs at the transcriptional and post-transcriptional levels [9]. In the past decade it has been shown that microRNAs (miRNAs) are important components of post-transcriptional fine tuning of gene expression in mammals [10–12].

miRNAs are short, 18–25-nucleotide-long, single-stranded, non-coding RNA molecules. They are transcribed from different regions of the genome, including intergenic and intronic/exonic regions of protein-coding genes, by RNA polymerase II. Primary transcripts are processed in two steps during miRNA biogenesis by the RNase III enzymes Drosha and Dicer [13]. The mature miRNAs are incorporated into the RNA-induced silencing complex (RISC) [11] and generally bind the 3′ untranslated region (3′ UTR) of target messenger RNAs (mRNAs) and act as negative regulators of gene expression through inhibition of protein synthesis and/or induction of target mRNA degradation [10, 11]. Several miRNAs, such as miR-155, miR-21, and miR-146, are induced in macrophages in response to LPS, suggesting that they have a role in the regulation of macrophage activation and M1-type polarization [14–18]. In addition, a number of miRNAs have recently been linked to alternative macrophage activation. For instance, miR-125b-5p, miR-199b, and miR-378-3p showed elevated expression in nematode infection-elicited alternative macrophage activation [19]. Among these miRNAs, miR-378-3p has been shown to repress IL-4-driven macrophage proliferation through the modulation of the PI3K/AKT signaling pathway [19]. Furthermore, miR-124, miR-324-5p, and miR-511-3p have been shown to fine-tune macrophage functions during allergic inflammation and tumor development, conditions characterized by the predominance of alternative macrophage activation [20–23]. Finally, miR-193b has been found to be induced by IL-4 in human macrophages, although its function remains unknown [24].

Despite the fact that IL-4-induced alternative macrophage activation-specific miRNAs have been identified in human and mouse in in vitro as well as in vivo models [19, 24], the regulatory mechanisms of miRNA expression and their role during alternative macrophage activation remain poorly understood. In this work we identified the human alternative macrophage activation-specific microRNome using miRNA microarray and quantitative PCR (qPCR) techniques. We found IL-4-dependent induction of miR-342-3p and miR-193b and repression of miR-99b and miR-125a-5p. Intriguingly, IL-4-dependent regulation of miR-342-3p, miR-99b, and miR-125a-5p is conserved between human cells and mouse bone marrow-derived macrophages. Furthermore, these miRNAs show the same expression pattern in an in vivo mouse model of parasitic infection. The IL-4-regulated expression of miR-342-3p, miR-99b, and miR-125a-5p is dependent on IL-4Rα and STAT6 expression as demonstrated by loss of function genetic experiments. Additionally, miR-342-3p and its host gene, Evl (Ena/Vasp-like), are co-regulated during IL-4-induced alternative macrophage activation in mouse. Chromatin immunopreciptiation (ChIP) confirmed that STAT6 directly binds to the regulatory region of the EVL gene in IL-4-stimulated human and mouse macrophages. Functional analyses combined with miRNA manipulation studies demonstrated that miR-342-3p decreases the viability of macrophages by inducing apoptosis. In silico target prediction and functional analyses identified the anti-apoptotic direct target gene network of miR-342-3p, which includes Bcl2l1. These findings suggest that enhanced miR-342-3p expression is a component of a negative feedback loop controlling excessive macrophage proliferation during Th2-type inflammatory responses.

## Methods

### Cell isolation and culture

Isolation of human monocytes, mouse bone marrow cells, and mouse peritoneal macrophages was done as described earlier with minor modifications [19, 25, 26]. CD14^+^ human monocytes were isolated from platelet-free buffy coats from healthy donors by Ficoll gradient centrifugation followed by immunomagnetic cell separation with anti-CD14-conjugated microbeads (VarioMACS Separation System, Miltenyi Biotec.). Monocytes were cultured in multiwell culture plates in RPMI 1640 (Sigma-Aldrich) supplemented with 10 % fetal bovine serum (Invitrogen) and penicillin/streptomycin (Sigma-Aldrich). For macrophage differentiation and alternative macrophage activation, freshly plated monocytes were treated with IL-4 (100 ng/ml; PreproTech). Cells were harvested 12 and 72 h after cytokine treatment. Bone marrow isolation and bone marrow-derived macrophage (BMDM) differentiation were performed as described before [26]. Bone marrow was flushed from the femur of wild-type (WT), IL-4Rα, and STAT6 knockout (KO) male animals. Cells were purified through a Ficoll-Paque gradient (Amersham Biosciences, Arlington Heights, IL, USA) and cultured in DMEM containing 20 % endotoxin-reduced fetal bovine serum and 30 % L929 conditioned medium for 5 days. For alternative macrophage activation we treated freshly isolated bone marrow cells with IL-4 (5 ng/ml; PreproTech). Culture medium was changed for Macrophage SFM medium (Gibco) on the 5th day. IL-4 (20 ng/ml) was further added to the culture of macrophage colony stimulating factor (MCSF)- and IL-4-pretreated macrophages for 24 h for the alternative macrophage activation. Peritoneal exudate cells from *Brugia malayi*-infected animals were seeded at 5 × 10^6^ cells per well to six-well cell culture plates (NUNC) in RPMI, 5 % fetal calf serum, 2 mM L-glutamine, 0.25 U/mL penicillin, and 100 mg/mL streptomycin. After 4 h of incubation at 37 °C, 5 % CO_2_, non-adherent cells were washed off and the adherent cells were lysed in 700 μL Qiazol.

### Animals and infection

Wild-type mice (WT; C57BL/6J or BALB/c), Stat6-deficient (Stat6 KO) mice on C57BL/6 J, and IL-4 receptor-α-deficient (IL-4Rα KO) mice on a BALB/c background were housed under minimal disease conditions. Surgical implantation of adult *B. malayi* parasites into WT (C57BL/6) mice was carried out as described previously [19] and performed by the same group and at the same facilities as in case of of the results presented by Jenkins et al. [27].

### Flow cytometry

Macrophages were resuspended in staining medium (phenol-red free DMEM, 10 mM HEPES, 2 % fetal bovine serum) and incubated with anti-mouse CD206 (AbD Serotec), anti-human CD206 (BD Biosciences), or corresponding unspecific isotype control antibodies for 20 minutes at 4 °C. Cells were washed and resuspended in staining medium for flow cytometry. Data acquisition was performed using a FACSAria III instrument (BD Biosciences) and data were analyzed with FlowJo software.

### Arginase activity

Arginase activity measurement was performed as previously described with minor modifications [28].

### miRNA microarray and data analysis

Global miRNA expression data were obtained with Affymetrix miRNA 1.0 arrays (Affymetrix). Total RNA samples were labeled using an Affymetrix FlashTag Biotin HSR RNA Labeling Kit according to the manufacturer’s protocol. Briefly, 500 ng of total RNA samples were poly(A)-tailed using poly A polymerase enzyme and ATP at 37 °C for 15 minutes, then biotinylated by ligating biotin-labeled fragment to the 3′ end. Labeled samples were hybridized on miRNA 1.0 arrays at 48 °C and 60 rpm for 16 h. After that, arrays were washed and stained using the standard Affymetrix protocol with an Affymetrix Hybridization, Wash, and Stain Kit on a FS 450 fluidic station instrument. The arrays were scanned using an Affymetrix GeneChip Scanner 3000 7G instrument.

The miRNA CEL files were processed with R, using affy and mirna10cdf packages [29, 30]. Raw expression values were processed with the robust multi-array average (RMA) procedure (background adjustment, quantile normalization, and summarization of log-expression values for each miRNA for each array) [31]. Only probes annotated as human miRNAs were kept. Lowly expressed probes were removed if expression was lower than the median in at least two samples. The interquartile range was calculated across all samples for each probe to determine the most variable probe of those mapped to the same miRNA; 563 probes were annotated, expressed, and unique. Quality control was performed using principal component analysis. Differential expression analysis was performed using the limma package [32, 33] by fitting a linear model for each probe then using an empirical Bayes method to get moderated t-statistics. The Benjamini and Hochberg method was used to calculate false discovery rates (FDRs) [34].

### Small RNA-Seq and data analysis

A cDNA library for small RNA-Seq was generated from 1 μg total RNA using an Illumina TruSeq Small RNA Sample Preparation kit, according to manufacturer’s protocol. Briefly, after ligation of 3′ and 5′ RNA adapters, reverse transcription was performed to synthesize cDNA; then the cDNA was amplified using a common primer and primer-containing index sequence. The amplified product/library was excised from gel (6 % Novex TBE PAGE gel) and, after purification, the libraries were quantified by Qubit fluorometer and checked on a BioAnalyzer 2100 using DNA1000 chip (Agilent Technologies). The small RNA-Seq libraries were diluted to 10 nM and five libraries were pooled together before sequencing. A single-read 50-bp sequencing run was performed on an Illumina HiScan SQ instrument (Illumina, San Diego, CA, USA). Each library pool were sequenced in one lane of a sequencing flow cell; 16–18 million reads per sample was obtained. Sequencing results from small RNA libraries were 3′ adapter trimmed and de-duplicated using the *reaper* and *tally* command-line tools from Kraken [35]. The processed small RNA-Seq reads were mapped to all available *Mus musculus* mature miRNA sequences (miRBase v21 [36]). Read mapping was performed using Bowtie [37]. The mapping process for reads longer than ten nucleotides allowed two mismatches. Quantification of mapped reads with minimum and maximum lengths of 18 and 24 nucleotides overlapping mature miRNA regions (miRBase v21) was performed using the R package GenomicRanges [38, 39]. The minimum overlap between mapped reads and mature regions was 16. We filtered out mature miRNAs with counts per million (cpm) lower than or equal to 4 cpm across at least three libraries. Differential expression analysis was performed using the edgeR package [40] fitting a negative binomial generalized linear model for each mature miRNA and then the likelihood ratio test was performed. The comparisons were: WT in the presence of IL-4 versus WT control, WT in the presence of IL-4 versus STAT6 KO in the presence of IL-4, STAT6 KO in the presence of IL-4 versus STAT6 KO control, and WT control versus STAT6 KO control. The Benajmini and Hochberg method was used to calculate FDRs [34].

### Real-time qPCR

Total RNA was isolated from cells using Tri Reagent (MRC) according to the manufacturer’s protocol. For quantification of mRNAs, pri-miRNAs, and mature miRNAs, reverse transcription was performed using a High-Capacity cDNA Reverse Transcription Kit (Applied Biosystems). Reverse transcription (RT) primers for mature miRNAs were supplied by Applied Biosystems. Transcript quantification was performed by quantitative real-time RT-PCR using Taqman probes or SYBR Green assays (selfmade assays) and Taqman Gene Expression Assays (Applied Biosystems).

For semi-quantitative PCR-based quantification of miR-125a_Ncrna00035 in unstimulated and IL-4-stimulated macrophages, specific primers were designed with Primer3. The PCR program used for semi-quantitative amplification was: 95 °C for 10 minutes, followed by 25 cycles of 30 s at 95 °C, 60 s at 65 °C, and 30 s at 72 °C. PCR products were separated by agarose gel electrophoresis and visualized by GelRed staining. qPCR assays and primer sequences are listed in Additional file 1.

### GRO-seq and ChIP-seq data analysis

All samples were aligned to the mouse reference genome (mm9) [41] and RNA-Seq data were analyzed using the standard TopHat/Cufflinks pipeline [42]. For ChIP-seq and GRO-seq (global run-on sequencing) data an integrative analysis pipeline was applied [43]. Data were visualized in Integrated Genome Browser [44].

### Chromatin immunopreciptiation

ChIP was performed as previously described with minor modifications [45]. Briefly, cells were crosslinked with DSG (Sigma) for 30 minutes and then with formaldehyde (Sigma) for 10 minutes. After fixation, chromatin was sonicated with a Diagenode Bioruptor to generate 200–1000-bp fragments. Chromatin was immunoprecipitated with antibodies against pre-immune IgG (Millipore, 12–370), H3K27ac (Abcam, ab4729) and STAT6 (Santa Cruz, sc-981). Chromatin–antibody complexes were precipitated with Protein A-coated paramagnetic beads (Life technologies). After six washing steps, complexes were eluted and reverse crosslinked. DNA fragments were column purified (Qiagen, MinElute). The amount of immunoprecipitated DNA was quantified with a Qubit fluorometer (Invitrogen). DNA was applied for qPCR analysis. Primer sequences are listed in Additional file 2.

### Transient transfection

To determine the potential function of miR-342-3p, RAW264.7 cells were transfected with 30 nM miR-342-3p precursor (Applied Biosystems) or scrambled miRNA negative control (Applied Biosystems) using DharmaFECT 3 (Thermo Scientific) in 12- and 96-well plates (Sigma-Aldrich) and eight-well chamber slides (Thermo Scientific).

### mRNA microarray and computational target identification

The raw sample CEL files were processed within R using the affy, org.Mm.eg.db and mogene10stv1cdf packages [29, 46, 47] Similarly to the miRNA microarray processing, raw expression values were processed with the RMA procedure [31]. The interquartile range was calculated across all samples for each probe in an identical way to the miRNA microarray methodology to remove probes mapping to the same transcript. Quality control was performed using principal component analysis. Differential expression analysis was performed as mentioned in the “miRNA microarray and data analysis” section, using the limma package [32] to fit a linear model to each probe then using an empirical Bayes method to determine moderate t-statistics. The comparison used was transfection with miR-342-3p versus miR-negative control miRNA mimics in the mouse macrophage cell line RAW264.7. The Benjamini and Hochberg method was used to calculate FDRs [31]. The comparison was used to verify direct effects due to miR-342-3p regulation. Sylamer [48] was used to quantify the enrichment or depletion of miRNA seed matches in the 3′ UTRs of genes. The genes with enriched 3'UTRs were ordered according to the t-statistic (differential expression). Prediction of functional miRNA targets was performed by TargetExpress (C Ovando-Vázquez, D Lepe-Soltero, C Abreu-Goodger, submitted). TargetExpress is a support vector machine combination based on TargetScan [49] and microT-CDs [50] predictions and an expression profile. In this case, the miR-negative control expression profile was included in the TargetExpress model.

The 3′ UTR sequences corresponding to selected downregulated and computationally predicted anti-apoptotic mmu-miR-342-3p target genes were obtained using the TxDb.Mmusculus.UCSC.mm10.ensGene and GenomicFeature packages [38, 51] Conservation scores for these 3′ UTR sequences were obtained from the UCSC GenomeBrowser using the rtracklayer package [52], selecting the track cons60way to obtain the phastCons60wayPlacental table.

### Cell number analysis

Propidium iodide (PI)-based cell number analysis was performed as previously described with minor modifications [53]. Briefly, macrophages attached to the bottom of 96-well plates were permeabilized by Triton X-100, stained by PI and measured in a Synergy HT microplate reader (Bio-Tek Instruments, Winooski, VT, USA) at 530/25 nm excitation and 645/40 nm emission. Cell numbers were determined using the cell number reference curve.

### Cell viability analysis

Resazurin-based viability staining (Promega) was carried out according to the manufacturer’s protocol. Neutral red staining was performed as previously described with minor modifications [54].

### Cell cycle analysis

We plated 2 × 10^4^ cells per well in Labtek 8-well chambered coverslips and transfected them with appropriate miRNAs. Cell fixation and PI staining were performed 48 h post-transfection as previously described [55] with minor modifications. Raw264.7 cells were fixed on chamber slides using −20 °C methanol at −20 °C overnight. Fixed cells were covered with 5 μg/ml PI (Sigma-Aldrich) and 200 μg/ml RNase A (Sigma-Aldrich) in 0.4 ml phosphate-buffered saline for 2 h at room temperature in the dark. Measurements were performed on a slide-based iCys Research Imaging Cytometer (CompuCyte Corporation, Westwood, MA, USA). The DNA dye was excited by the 488 nm laser line and emission was collected in the red channel with a 650 nm longpass filter on a linear scale. Single cells were recognized according to their size and circularity and cells were further gated according to their integral fluorescence intensity and maximum pixel intensity on a two-dimensioanl scattergram of PI signal. Data from the one-dimensional histogram of the DNA staining was exported and fitted with the automatic one cycle diploid model of the Modfit LT 3.0 software (Verity Software House) with AutoDebris compensation, AutoAggregate Compensation, and Apoptosis Model. In the measurements, the G1–G2 linearity ratio was around 1.8 and the RCS (Reduced Chi-Square, a measure of goodness of fit) was less than 5. Measurements were repeated three times and approximately 1000–2000 cells were collected from each well.

### Apoptosis assay

Raw264.7 cells were plated and treated in a similar manner as for the cell cycle analysis. Annexin V-FITC/PI staining was performed as previously described [56] with minor modifications. Forty-eight hours following miRNA transfection, the culture medium was replaced by 100 μl AB buffer (140 mM NaCl and 2.5 mM CaCl_2_), 10 μl Hoechst 33342 solution (50 μg/ml stock), and 10 μl propidium iodide solution (50 μg/ml stock) and 5 μl FITC-conjugated annexin V (AV) was added (according to the description of the MBL Apoptosis Detection Kit) for 15 minutes at 37 °C. Laser-scanning cytometric measurements were made using a iCys Research Imaging Cytometer (CompuCyte Corporation, Westwood, MA, USA). Hoechst, which stains all cells, was excited at 405 nm and detected with a 463/20 bandpass filter; PI, which stains dead cells, was excited at 488 nm and detected with a 650 nm longpass filter; and the AV signal, which indicates apoptotic cells, was excited at 488 nm and detected with a 530/30 bandpass filter. Single cells were selected by size and circularity and additionally gated by their DNA content. Early apoptotic AV^+^/PI^−^, late apoptotic AV^+^/PI^+^, and unapototic AV^−^/PI^−^ cells were detected using a quadrant gate in the PI versus AV dot plot with respect to unlabeled and miRNA untreated cells.

### Plasmid construction

PsiCHECK2 dual luciferase vector (Promega) was used to confirm the function of the putative miR-342-3p binding sites in the Bcl2l1 3′ UTR. For luciferase reporter assays, 320 bp (miR-342-3p_1) and 309 bp (miR-342-3p_2) of the 3′ UTR of the Bcl2l1 gene, including the mir-342-3p target sites, were amplified by PCR using F1/R1 and F3/R3 primer pairs with XhoI and NotI sites. PCR was performed on mouse macrophage-derived genomic DNA. The XhoI/NotI-digested PCR product was cloned into the XhoI/NotI-digested psiCHECK2 dual luciferase vector. F1/R2 and F2/R1 as well as F3/R4 and F4/R3 primers were used to delete the mir-342-3p target sites from the 3′ UTR. After mixing the two PCR products and digestion with XhoI and NotI, the 3′ UTR fragment with deleted mir-342-3p binding sites was cloned into a XhoI/NotI-digested psiCHECK2 vector. Primer sequences are given in Additional file 2.

### Luciferase assay

Luciferase assays were performed as described previously [57]. Brifely, HEK293 cells were grown on 24-well plates to 70–80 % confluence. Transfection was performed using PEI with 0.1 μg of pRL-TK (Rr-luc) containing 3′ UTR sequences and 0.1 μg of pGL3 control vector (Pp-luc) (Promega) for either a specific miR-342 or small interfering RNA control. Cell lysates were collected 30–36 h post-transfection and luciferase activity was determined using a Dual-Luciferase® Reporter Assay System (Promega, Mannheim, Germany). Renilla luciferase activity was normalized to firefly luciferase activity for each cell culture well and averaged across six well repetitions per condition.

### Statistical analysis

The two-tailed Student’s *t*-test was used to evaluate the significance of the differences between two groups; *p* values <0.05 were considered significant.

## Results

### Identification of the human alternatively activated macrophage-specific miRNA signature

**Fig. 1:**
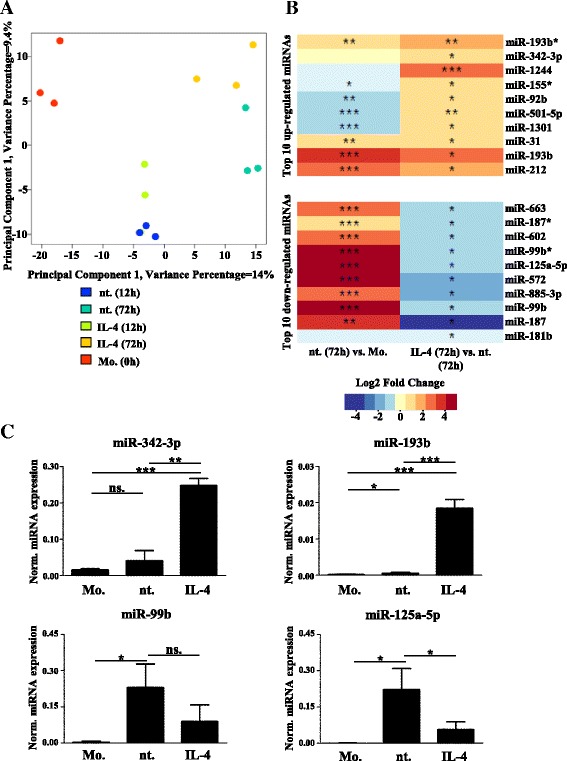
The IL-4 regulated microRNome in human alternatively activated macrophages. **a** Two-dimensional principle component analysis of the miRNA transcriptome of human peripheral blood-derived monocytes, as well as unstimulated and IL-4-stimulated differentiated macrophages obtained from three independent donors. Principal component analysis was carried out on all miRNAs expressed in monocytes and/or macrophages (500 miRNAs). Color scheme: monocytes (*Mo.*), *red*; 12-h nontreated (*nt.*) differentiating macrophages, *blue*; 12-h IL-4 stimulated differentiating macrophages, *light green*; 72-h nontreated macrophages, *dark green*; and 72-h IL-4-stimulated macrophages, *orange*. **b** Heatmap showing average fold changes and significance of the top ten IL-4 upregulated and downregulated miRNAs (FDR ˂0.1) in 72-h unstimulated macrophages compared with monocytes (*left panel*) and 72-h IL-4-treated macrophages compared with 72-h unstimulated macrophages (*right panel*) from three independent human donors. **c** Stem-loop RT-qPCR-based measurement of miR-342-3p, miR-193b, miR-99b, and miR-125a-5p expression in human monocytes, 72-h nontreated, and IL-4-stimulated macrophages. *Bars* show the mean ± standard deviation of normalized signal intensity values from four independent human donor-derived cells. *P ˂ 0.05, **P ˂ 0.01, ***P ˂ 0.001; *n.s.* not significant

In order to determine which miRNAs are expressed in human monocytes and differentiating macrophages in the presence of IL-4, CD14^+^ monocytes were separated from human peripheral blood and exposed to IL-4 for 12 or 72 h then subjected to microarray analysis in triplicate (the experimental design is shown in Additional file 3a). IL-4-induced expression of markers characteristic of alternative macrophage activation was confirmed by RT-qPCR and flow cytometry in human macrophages (Additional file 4a, b). Principal component analysis of microRNomes showed clear separation of monocytes from different stages of macrophage differentiation (Fig. 1a). In addition, alternative activation caused a moderate shift in the global macrophage microRNome at 72 h following IL-4 stimulation (Fig. 1a). Taken together, these results suggest that differentiation status and cytokine treatment correlate with distinct and characteristic miRNA expression profiles during human monocyte-derived macrophage differentiation. Next, we identified those miRNAs that were differentially regulated (FDR <0.1) 72 h after IL-4 treatment; 54 miRNAs showed significant IL-4-dependent regulation (33 upregulated and 21 downregulated; Fig. 1b; Additional file 5). We decided to further characterize two upregulated and two downregulated miRNAs. The selection was based on two criteria: (i) the miRNA expression level in IL-4-stimulated or control macrophages; and (ii) the published miRNA-associated functions. We examined miR-193b and miR-342-3p as two of the highly expressed IL-4-induced miRNAs. The IL-4-dependent induction of mir-193b expression is known, hence we used it as an adequate control of our differentiation method [24]. The other selected IL-4-enhanced miRNA, mir-342-3p, is associated with pro-apoptotic and anti-proliferative functions in different tumor types; it may, therefore, be a potential negative feedback regulator of IL-4-induced macrophage proliferation [11, 27, 58, 59]. Finally, we chose two members of the miR-99b-125a miRNA polycistron, including miR-99b and miR-125a-5p, where IL-4 was able to reduce the dramatic human monocyte–macrophage transition-dependent induction of miRNA expression. The expression changes of these miRNAs were validated using stem-loop RT-qPCR in monocytes as well as macrophages from four independent donors that were left unstimulated or differentiated with IL-4 (Fig. 1c; Additional file 6a). The expression of miR-342-3p was unchanged while miR-193b expression was slightly induced during the monocyte-to-macrophage transition, while both increased significantly in response to IL-4. In contrast, miR-99b and miR-125a-5p showed a significant induction during monocyte–macrophage differentiation. In addition, miR-99b and miR-125a-5p expression were attenuated in IL-4-stimulated macrophages in all four human donors, although the IL-4-dependent reduction of mir-99b expression did not reach statistical significance (*p* = 0.056) (Fig. 1c; Additional file 6a).

### Conserved IL-4Rα/STAT6 signaling-dependent regulation of miR-342-3p, miR-99b, and miR-125a-5p expression during in vitro and in vivo mouse alternative macrophage activation

**Fig. 2:**
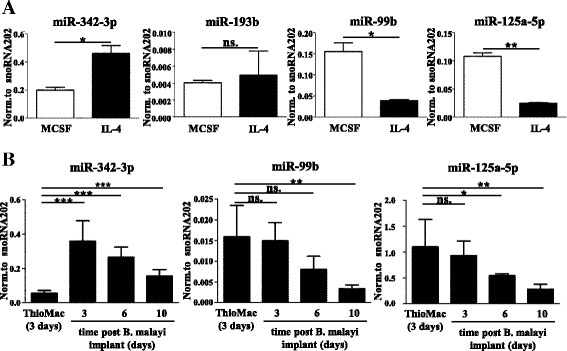
Conserved miRNA expression in mouse in vitro and in vivo differentiated and alternatively activated macrophages. **a** Stem-loop RT-qPCR-based measurement of miR-342-3p, miR-193b, miR-99b, and miR-125a-5p expression in IL-4-stimulated and unstimulated mouse bone marrow-derived macrophages. Each data point represents the mean and standard deviation of three individual animals. *P ˂ 0.05, **P ˂ 0.01; *ns.* not significant. **b** Stem-loop RT-qPCR-based quantification of miR-342-3p, miR-99b, and miR-125a-5p in mouse thioglycolate-elicited and in vivo alternatively activated macrophages. Each data point represents the mean and standard deviation of five individual animals. *P ˂ 0.05, **P ˂ 0.01, ***P ˂ 0.001; *ns.* indicates not significant

Next we asked if the identified IL4-dependent miRNAs (Fig. 1c) were regulated in an evolutionarily conserved manner between human and mouse. We therefore measured their expression in mouse bone marrow-derived macrophages (BMDMs) undergoing alternative macrophage activation using stem-loop RT-qPCR (the experimental design is shown in Additional file 3b). Alternative activation of mouse BMDMs was confirmed by flow cytometry and RT-qPCR-based measurements of known murine alternative macrophage activation markers and by assessment of arginase activity (Additional file 4c–e). As shown in Fig. 2a, miR-342-3p, miR-99b, and miR-125a-5p were regulated by IL-4 similar to the human cells. In contrast, miR-193b expression did not change upon IL-4 treatment. These findings suggest that the IL-4-mediated regulation of miR-342-3p, miR-99b, and miR-125a-5p is conserved between humans and mice.

**Fig. 3:**
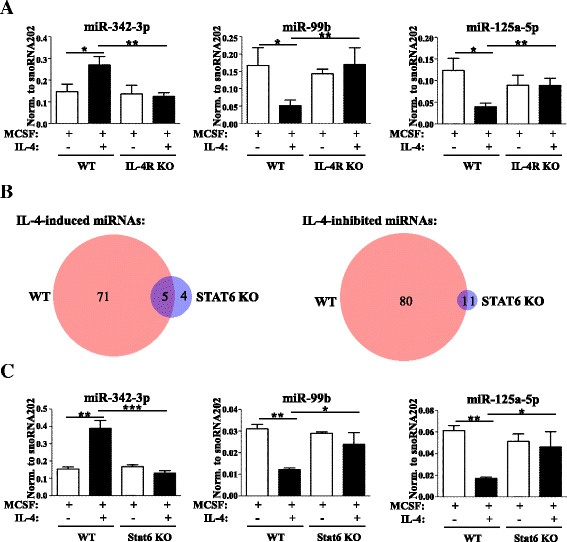
IL-4Rα and Stat6-dependency of IL-4-regulated miRNA expression in mouse macrophages. **a** miR-342-3p, miR-99b, and miR-125a-5p expression in IL-4-stimulated or unstimulated wild-type (*WT*) and IL-4Rα-defficient (*IL-4Rα KO*) mouse bone marrow-derived macrophages as measured by stem-loop RT-qPCR. Each data point represents the mean and standard deviation of four or three (WT or IL-4Rα KO, respectively) individual animals. *P ˂ 0.05, **P ˂ 0.01, ***P ˂ 0.001. **b** The number of miRNAs showing significant (FDR ˂0.1) IL-4-dependent regulation in wild-type (*WT*) and Stat6-deficient (*STAT6 KO*) macrophages. **c** Stem-loop RT-qPCR-based quantification of miR-342-3p, miR-99b, and miR-125a-5p expression in IL-4-stimulated or unstimulated WT and Stat6 KO mouse bone marrow-derived macrophages. Each data point represents the mean and standard deviation of three individual animals. *P ˂ 0.05, **P ˂ 0.01, ***P ˂ 0.001

In order to determine if the IL-4-mediated changes of miRNA expression measured in vitro also occur in vivo, we applied a mouse model of alternative macrophage activation. As described previously, intraperitoneal implantation of the filarial nematode *B. malayi* results in the accumulation of macrophages with M2-like characteristics, including elevated expression of Ym1, Fizz1/RELM-α, and Arg1 [7, 60]. Based on this, we determined miR-342-3p, miR-99b, and miR-125a-5p expression in nematode-elicited macrophages at different time points after infection (the experimental design is shown in Additional file 3c). In line with our in vitro results, miR-342-3p expression was upregulated in nematode-elicited macrophages 3 days after *B. malayi* implantation compared with naïve cells and showed continuously decreasing kinetics at later time points (Fig. 2b). Furthermore, both miR-99b and miR-125a-5p showed reduced expression during *B. malayi*-induced alternative macrophage activation, also reinforcing our in vitro findings (Fig. 2b). These results suggest that the conserved IL-4-dependent regulation of miR-342-3p, miR-99b, and miR-125a-5p expression holds in vivo relevance. Therefore, we focused our efforts on the identification of the upstream regulator(s) of miR-342-3p, miR-125a, and miR-99b expression during mouse alternative macrophage activation. In macrophages, IL-4 acts via IL-4Rα-containing type I and II receptor complexes [6]. As expected, we found that both the IL-4-mediated induction of miR-342-3p as well as the reduction of miR-99b and miR-125a-5p expression were completely abolished in IL-4Rα-deficient macrophages, confirming the requirement of the receptor for transmitting the IL-4 stimulus (Fig. 3a). IL-4 receptor complexes activate both the JAK/STAT6 and PI3K signaling pathways [6]. Since STAT6 transcription factor has been shown to be the dominant mediator of IL-4-induced transcriptional changes in macrophages [6], we hypothesized that STAT6 might be primarily responsible for the IL-4-induced changes in the macrophage microRNome. In order to assess the contribution of STAT6 to the regulation of the IL-4-responsive miRNAs, we analyzed the microRNome of WT and STAT6-deficient macrophages in the absence and presence of IL-4. We found that the majority of IL-4-responsive miRNAs in WT macrophages were STAT6-dependent (151 from 157; Fig. 3b; Additional file 7). Interestingly, we found only 11 miRNAs that showed changed expression upon IL-4 stimulus in STAT6-deficient macrophages (Fig. 3b; Additional files 7 and 8a). The IL-4-induced upregulation of miR-342-3p and downregulation of miR-99b and miR-125a-5p proved to be STAT6-dependent, which was also validated by stem-loop RT-qPCR (Fig. 3c). Taken together, these findings suggest that these miRNAs are regulated exclusively via the IL-4Rα/STAT6 signaling axis during alternative macrophage activation.

### Direct Stat6-dependent induction of miR-342-3p and its host gene, EVL, during alternative activation of murine and human macrophages

Intronic miRNAs and their host genes are often co-regulated, although there are known instances when intronic miRNAs are regulated by their own promoter region independently of that of the host gene [61, 62]. miR-342-3p is encoded within the third intron of the EVL gene in humans and mice and its expression showed coordinated regulation in both human colorectal cancer and multiple myeloma [63, 64]. Therefore, we decided to further characterize the potential regulatory mechanism controlling the expression of the host gene and the miRNA during human and mouse alternative macrophage activation. For this reason we analyzed publicly available GRO-seq data from unstimulated and ChIP-seq datasets from both IL-4-stimulated and unstimulated mouse macrophages [65, 66].

**Fig. 4:**
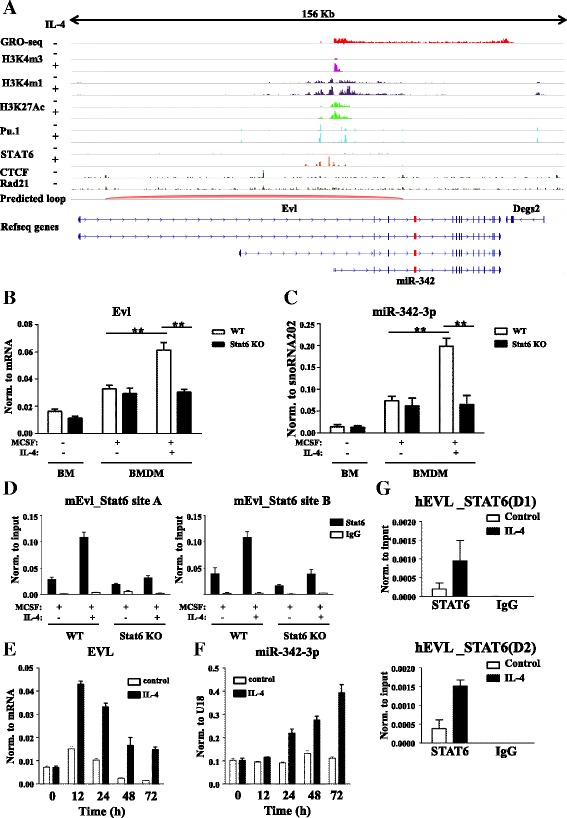
Mechanism of IL-4-dependent co-regulation of miR-342-3p and EVL expression in human and mouse macrophages. **a** Strand-specific GRO-Seq, CTCF, and Rad21-specific ChIP-seq signals in unstimulated as well as H3K4m3, H3K4m1, H3K27Ac, Pu.1, and Stat6 ChIP-Seq signals in IL-4-stimulated and unstimulated mouse macrophages at the EVL locus visualized by the Integrative Genomics Viewer. **b** RT-qPCR-based measurement of Evl expression in bone marrow (*BM*) cells and IL-4 stimulated or unstimulated bone marrow-derived macrophages (*BMDM*) in wild-type (*WT*) or Stat6-deficient (*Stat6 KO*) mice. Each data point represents the mean and standard deviation (SD) of three individual animals. **P ˂ 0.01. **c** Stem-loop RT-qPCR-based quantification of miR-342-3p expression in bone marrow cells and IL-4 stimulated or unstimulated bone marrow-derived macrophages in WT or Stat6 KO mice. Each data point represents the mean and SD of three individual animals. **P ˂ 0.01. **d** IL-4-induced Stat6 binding of two regulatory regions of the mouse EVL gene in WT and Stat6 KO mouse macrophages measured by ChIP-qPCR. Columns represent mean arbitrary units described in the “Methods” section ± SD. **e** RT-qPCR-based measurement of EVL expression during human macrophage differentiation in the absence or presence of IL-4 (a representative example of three independent human donors is shown). **f** Stem-loop RT-qPCR-based quantification of miR-342-3p expression during human macrophage differentiation in the absence or presence of IL-4 (a representative example of three independent human donors is shown). **g** IL-4-induced recruitment of STAT6 to the human EVL (*hEVL*) locus in macrophages obtained from two independent human donors (*D1* and *D2*) measured by ChIP-qPCR. Data are expressed as mean ± SD

Combined analysis of transcription of nascent RNA (GRO-seq) and the location of the active transcription start site (TSS)-specific histone mark H3K4m3 (ChIP-seq) data showed that the shortest known transcript variant of Evl (NM_001163396) was transcribed in resting mouse macrophages (Fig. 4a). Furthermore, the H3K4m3 histone modification was increased at the TSS of Evl in IL-4-stimulated macrophages compared with unstimulated cells (Fig. 4a). Interestingly, the H3K4m3 peak was not detected in the intronic region of Evl around the miR-342-3p coding region, suggesting that Evl and miR-342-3p utilized a common TSS in resting and alternatively activated mouse macrophages (Fig. 4a). To further explore the IL-4-dependent regulation of miR-342-3p and Evl in mice, we measured the expression of both mature miRNA and its host gene in mouse bone marrow cells as well as in IL-4-stimulated and unstimulated BMDMs. Both Evl and miR-342-3p expression levels were induced during mouse BMDM differentiation, which was further increased by IL-4 in a STAT6-dependent manner (Fig. 4b, c).

Moreover, we examined the macrophage lineage-specific transcription factor Pu.1 and IL-4-activated STAT6 binding at the predicted EVL-associated functional domain/chromatin loop. First, CTCF and Rad21 ChIP-seq data sets from unstimulated macrophages were used for the functional domain/chromatin loop prediction. Functional chromatin domains were defined using the following criteria: (i) common CTCF and Rad21 binding at the starting points of chromatin interactions; and (ii) a predominantly convergent direction of the CTCF elements under CTCF/Rad21 co-peaks [67–70]. One distinct Evl-associated putative functional domain (loop) was predicted using these features (Fig. 4a). Next, Pu.1-bound genomic regions were identified and examined within the predicted functional domain in both unstimulated and IL-4-treated macrophages but Pu.1-binding was not influenced by IL-4 stimulation (Fig. 4a). In addition, IL-4-induced Stat6 peaks were detected both upstream and downstream of the TSS of the Evl gene in mouse macrophages [65] (Fig. 4a).

Next we validated two Stat6-bound genomic regions −4 kb (mEvl_Stat6 site A) and +4 kb (mEVL_Stat6 site B) from the TSS. ChIP followed by qPCR showed an enrichment of Stat6 binding at these sites in IL-4-stimulated WT macrophages relative to untreated WT and IL-4-treated Stat6-deficient macrophages as well as IgG controls (Fig. 4d). These are the likely enhancers controlling Evl as well as miR-342-3p induction. In order to determine whether IL-4-mediated co-regulation of EVL and miR-342-3p is conserved between human and mouse, we measured their expression in human monocyte-derived unstimulated and IL-4-stimulated macrophages. As shown in Fig. 4e and Additional file 6b, IL-4 induced EVL expression in all three donors after 12 h, which then was reduced at later time points. miR-342-3p followed similar but delayed kinetics upon IL-4 treatment as its elevated expression was only detectable after 24 h (Fig. 4f; Additional file 6c). In order to assess whether STAT6 binds the genomic locus of human EVL, we utilized public human STAT6 ChIP-seq datasets from IL-4 stimulated Th2-type T cells [71]. Interestingly, a genomic region within +0.3 kb of the TSS of the EVL gene was occupied by STAT6 in these cells. Therefore, we hypothesized that this region might also be occupied by STAT6 in human alternatively activated macrophages. This could be confirmed by ChIP-qPCR in two independent donors (D1 and D2; Fig. 4g). These findings suggest that miR-342-3p and its host gene, EVL, are coordinately regulated by IL-4 via direct DNA binding of STAT6 in both mouse and human macrophages.

### Alternative macrophage activation-specific repression of the miR-99b-125a miRNA polycistron is mediated at the transcriptional level by IL-4/STAT6 signaling

**Fig. 5:**
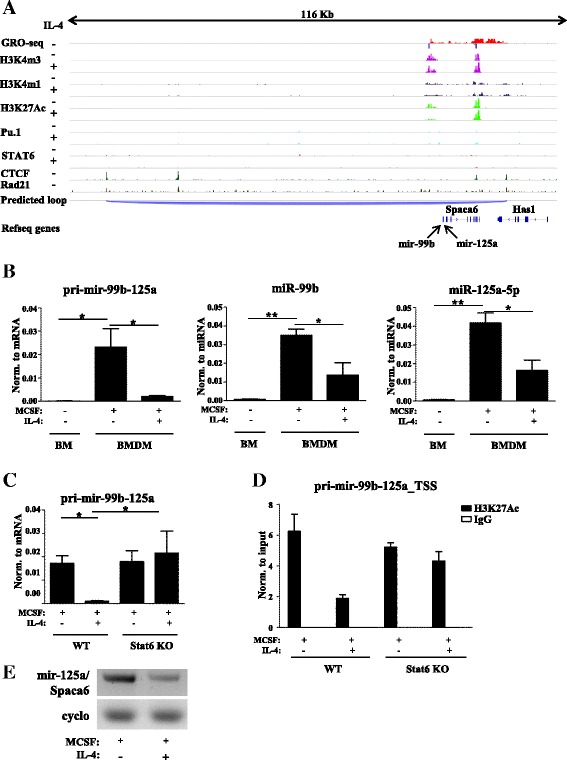
IL-4-dependent and Stat6-mediated repression of the miR-99b-125a miRNA polycistron. **a** Strand-specific GRO-Seq, CTCF, and Rad21-specific ChIP-seq signals in unstimulated as well as H3K4m3, H3K4m1, H3K27Ac, Pu.1, and Stat6 ChIP-Seq signals in IL-4-stimulated and unstimulated mouse macrophages at the miR-99b-125a and Spaca6 loci visualized by the Integrative Genomics Viewer. **b** Pri-miR-99b-125a, miR-99b, and miR-125a-5p expression in bone marrow cells (*BM*), MCSF, and MCSF + IL-4-treated BMDMs. Each data point represents the mean and standard deviation (SD) of three individual animals. *P ˂ 0.05, **P ˂ 0.01. **c** Expression of the pri-miR-99b-125a polycistron in IL-4-stimulated or unstimulated BMDMs derived from wild-type (*WT*) or Stat6-defficient (*Stat6 KO*) mice. Each data point represents the mean and SD of three individual animals. *P ˂ 0.05. **d** H3K27Ac ChIP-qPCR in IL-4-treated or nontreated WT or Stat6 KO mouse BMDMs at the potential TSS of pri-miR-99b-125a. Data are expressed as mean ± SD. **e** RT-PCR-based detection of a common transcript of pri-miR-99b-125a and Spaca6 in IL-4-treated or nontreated WT mouse BMDMs. Cyclophilin A (cyclo) serves as normalization control

Similarly to the analyses described above (Fig. 4a), we took advantage of publicly available GRO-seq datesets from unstimulated as well as ChIP-seq datasets from IL-4-stimulated and unstimulated mouse macrophages [65] to identify the primary transcripts coding miR-99b and miR-125a and further investigate the regulation of miR-99b and miR-125a expression. We identified only one overlapping H3K4m3 peak with a divergent GRO-seq signal, a hallmark of an active enhancer, downstream of the miR-99b and miR-125a-coding genomic region, confirming the common TSS and polycistronic transcription of miR-99b and miR-125a (Fig. 5a). In order to determine whether IL-4 represses miR-99b and miR-125a expression at the transcriptional or mRNA maturation level, we measured the expression of both primary (pri-miR-99b-125a) and mature transcripts in mouse bone marrow as well as IL-4-stimulated or unstimulated BMDMs (the genomic localization of the pri-miR-99b-125a-specific primer pair is shown in Additional file 9). miR-99b, miR-125a-5p, and pri-miR-99b-125a expression levels were increased to a similar extent in BMDMs compared with the bone marrow cells (Fig. 5b). Furthermore, IL-4 was able to reduce the expression of both pri-miR-99b-125a and the mature miRNAs in BMDMs compared with untreated cells (Fig. 5b). As shown in Fig. 3b, IL-4-mediated negative regulation of mature miR-99b and miR-125a-5p expression proved to be Stat6-dependent in mouse macrophages; hence, it was logical to assume that IL-4 represses the primary miRNA in a similar manner. Therefore, the expression level of pri-miR-99b-125a was measured in IL-4-stimulated and unstimulated BMDMs derived from WT and Stat6-deficient mice. We found that IL-4-dependent repression of pri-miR-99b-125a was completely abolished in the absence of Stat6 (Fig. 5c), suggesting that Stat6 is the key transcriptional mediator of the IL-4-dependent repression of miR-99b and miR-125a-5p expression. In order to determine the direct regulatory role of the lineage-specific transcription factor Pu.1 and the signal-specific transcription factor STAT6 in the IL-4-mediated repression of pri-miR-99b-125a expression, we sought to identify the Pu.1 and STAT6-bound genomic regions using resting and IL-4-stimulated, macrophage-derived, publicly available ChIP-seq dataset [65].

However, Pu.1- and Stat6-binding activity could not be detected within the predicted functional domain of pri-miR-99b-125a. These findings suggest that although the IL-4-mediated regulation of miR-99b and miR-125a expression is STAT6-dependent, (i) it might not be regulated via direct STAT6 DNA binding or (ii) the regulatory region might be outside of that explored within this study (Fig. 5a). Interestingly, H3K27 acetylation (the active histone mark) of the TSS of pri-miR-99b-125a was IL4-dependently reduced in WT but not in STAT6-deficient BMDMs (Fig. 5a, d), which further suggests that IL-4 represses transcription of the miR-99b-125a polycistron in a STAT6-dependent manner.

In addition, we found nascent RNA transcription at the genomic locus of the neighboring Spaca6 gene, although no H3K4m3 peak was detected at the 5′ end of this gene (Fig. 5a). Based on this observation, we hypothesized that the miR-99b-125a polycistron and Spaca6 might have common primary transcripts in macrophages. In order to test this, we used RT-PCR assays in which the PCR primers detected both miR-125a and the first predicted exon of Spaca6 (the genomic localization of a common pri-miR-99b-125a and Spaca6-specific primer pair is shown in Additional file 9). As shown in Fig. 5e, the expected 440-nucleotide PCR product was present in untreated macrophages and IL-4 treatment reduced the expression of this transcript (Fig. 5e). These findings suggest that the miR-99b-125a polycistron and Spaca6 form one STAT6-regulated transcription unit and they are encoded by the same primary transcript.

### miR-342-3p regulates cell proliferation and apoptosis-associated signaling pathways at the post-transcriptional level in macrophages

**Fig. 6:**
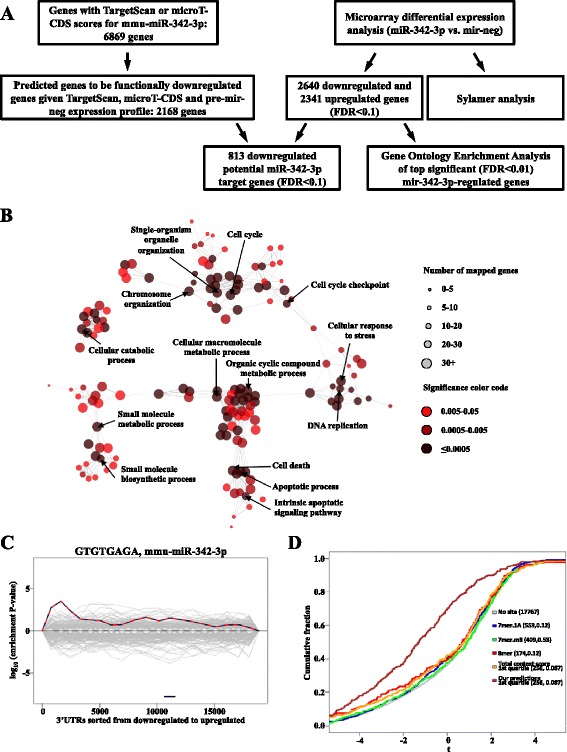
Global transcriptome and in silico analysis-based miR-342-3p target gene identification. **a** Schematic representation of combined microarray-based and computational miR-342-3p target gene identification. **b** Network visualization of Gene Ontology enrichment analysis of genes differentially expressed in miR-342-3p-transfected RAW264.7 mouse macrophages (FDR ≤0.01) using the ClueGO Cytoscape plugin. *Nodes* represent enriched GO biological process terms, *node colors* represent corresponding FDR values (Benjamini-Hochberg method), and *node sizes* represent the number of genes associated with the GO term. Only the label of the most significant term per group is shown. Nodes without second degree connections are omitted for clarity. **c** Sylamer analysis revealed that only miR-342-3p 8-mer *s*eed matches were enriched among downregulated genes in miR-342-3p-transfected macrophages compared with miR-negative control-treated cells. miR-342-3p and murine miRNA seed matches are represented as *red dashed lines* and *gray lines*, respectively. **d** Cumulative distributions of relative change (t-statistic) for different sets of potential miR-342-3p target genes with 7mer.1A (*blue*), 7mer.m8 (*green*), and 8-mer (*red*) seeds or the top 1000 TargetScan (*orange*) or microT-CDS (*purple*) and TargetExpress (*brown*) predictions to be functionally downregulated. Set size and the statistical significance of each set being more repressed than genes with no potential target site are shown in *parentheses*

In order to gain insights into the molecular pathways controlled by miR-342-3p, we examined the transcriptome of miR-342-3p-transfected RAW264.7 macrophages and identified a set of repressed miR-342-3p direct target genes using computational and biochemical approaches (a flowchart of experimental approaches is shown in Fig. 6a). Microarray analysis of miR-342-3p and miR-negative control-transfected cells identified 2640 downregulated and 2341 upregulated genes (FDR <0.1) in miR-342-3p-overexpressing macrophages compared with negative control-transfected cells (Fig. 6a; Additional file 10). To identify those biological functions whose activity might be regulated by miR-342-3p, we analyzed the list of the most significantly regulated genes (FDR ≤0.01) using the ClueGO Cytoscape plugin [72]. We found that the significantly overrepresented miR-342-3p-regulated Gene Ontology (GO) categories included cellular metabolism and regulation of viable cell number, including cell proliferation and cell death (Fig. 6b). In addition, Sylamer analysis of the microarrays derived from miR-342-3p and miR-negative control-transfected cells showed that the complementary sequence of the 8-mer seed region of miR-342-3p was enriched within the 3′ UTR of downregulated genes in miR-342-3p-transfected cells, confirming the specificity of the miR-342-3p-induced transcriptomic changes (Fig. 6c). For the identification of functionally important miRNA–mRNA interactions we used a new TargetExpress method (manuscript submitted; see “Methods”) that combines TargetScan [49] and microT-CDS [50] predictions with the gene expression profile of the negative control condition. The resulting predicted miR-342-3p targets showed the best correlation with miR-342-3p-dependent repression of gene expression in our experimental system (Fig. 6d); 813 predicted miR-342-3p target genes were downregulated in pre-miR-342-3p-transfected cells (Fig. 6a; Additional file 11). These findings raise the possibility that these downregulated genes are repressed directly by miR-342-3p. However, further experimental confirmation is required for the demonstration of direct miR-342-3p-dependent repression.

### miR-342-3p acts as a regulator of macrophage cell number via reduction of cell viability and induction of apoptosis

Our transcriptome analysis revealed cellular proliferation and cell death as major target processes regulated by miR-342-3p. Proliferation of local macrophage population is induced by IL-4-mediated Th2-type inflammation [27]. Furthermore miR-342-3p has been described to be aberrantly downregulated in different malignancies, including colorectal and breast cancers [58, 63]. In addition, overexpression of miR-342-3p was found to induce apoptosis and block cancer cell proliferation [59, 63]. Thus, we hypothesized that IL-4-induced miR-342-3p expression could be a potential negative feedback mechanism controlling excessive macrophage expansion upon Th2-type cytokine stimulus.

**Fig. 7:**
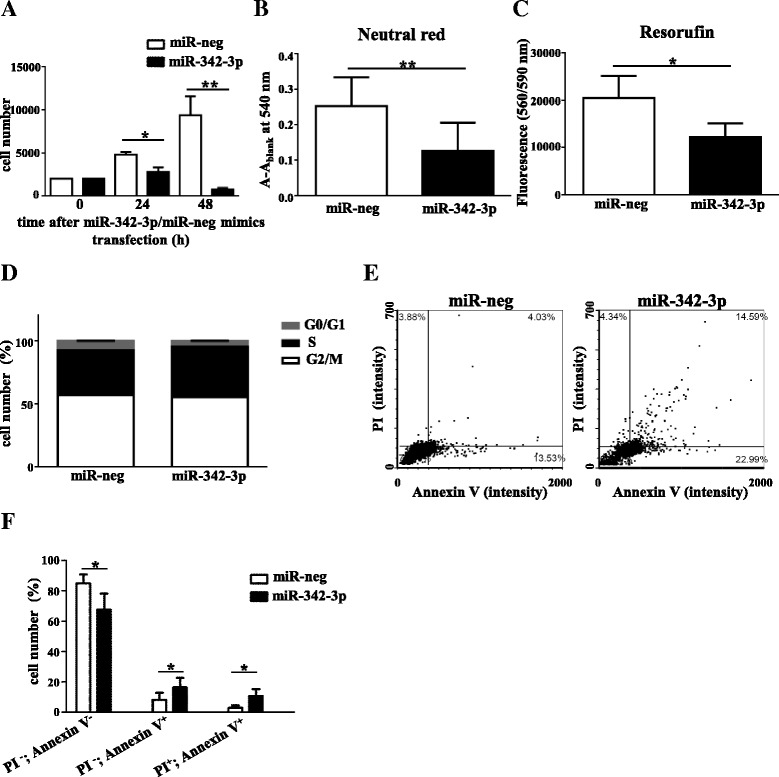
Reduced cell viability and increased macrophage apoptosis by miR-342-3p overexpression. **a** PI staining-based cell number analysis of RAW264. Seven cells at different time points following miR-342-3p mimic transfection. Each data point represents the mean and standard error of the mean (SEM) of five parallel samples from two independent experiments. *P ˂ 0.05, **P ˂ 0.01. **b** Neutral red-based cell viability analysis of miR negative control (*miR-neg*) and miR-342-3p (*miR-342-3p*) mimic-transfected RAW264.7 cells 48 h after miRNA mimic transfection. Each data point represents the mean and SEM of four independent experiments. *P ˂ 0.05. **c** Resorufin-based cell viability analysis of miR-negative control and miR-342-3p mimic-transfected RAW264.7 cells 48 h after miRNA mimic transfection. Each data point represents the mean and SEM of four independent experiments. *P ˂ 0.05. **d** Effect of miR-342-3p on cell cycle progression in RAW264.7 cells. Cell cycle distribution was evaluated by slide-based imaging cytometry after PI staining. Each data point represents the mean and standard deviation (SD) of three independent experiments. *P ˂ 0.05. **e** Representative dot plots of laser-scanning imaging cytometry analysis of AV- and PI-labeled miR negative control and miR-342-3p mimic-transfected RAW264.7 cells 48 h after miRNA mimic transfection. **f** Viable (PI-, AV-), early apoptotic (PI-, AV+) and late apoptotic (PI+, AV+) cell distribution 48 h following miR-negative control and miR-342-3p mimic transfection into RAW264.7 cells. Each data point represents the mean and SD of three independent experiments. *P ˂ 0.05

In order to test this hypothesis, we sought to explore the functional effect of miR-342-3p on macrophage proliferation and/or apoptosis in the mouse macrophage cell line RAW264.7 transfected with miR-342-3p or miR-negative control miRNA mimics. Following transfection, cell numbers were determined at various time points by propidium iodide (PI) staining of permeabilized adherent cells [53]. The cell number of miR-342-3p-overexpressing macrophages showed 40 and more than 80 % reduction at 24 and 48 h post-transfection, respectively, compared with the miR-negative control-transfected cells (Fig. 7a). To confirm our results, resazurin reduction and neutral red uptake-based assays as independent in vitro cell viability analyses were also carried out [54]. As shown in Fig. 7b, c, miR-342-3p reduced viable macrophage cell numbers by 40 and 55 % at 48 h post-transfection in the resazurin and neutral red uptake assays, respectively. In order to assess if the decreased cell viability observed is the result of impaired progression of the cells through the cell cycle or is due to increased cellular death, we performed cell cycle analysis by PI staining and a necrosis/apoptosis assay by annexin V (AV)/PI double-staining of the miR-342-3p and miR-negative control-transfected macrophages. As shown in Fig. 7d, analysis of cell cycle distribution by Hoechst staining and slide-based imaging cytometry revealed that miR-342-3p overexpression was associated with a slight but not significant increase in the number of cells in S phase. However, miR-342-3p overexpression significantly increased the number of both AV-positive/PI-negative early (Fig. 7e, lower right quadrants) and AV/PI double positive late (Fig. 7e, upper right quadrants) apoptotic cells (Fig. 7e, f). From these results we concluded that miR-342-3p regulates macrophage cell numbers via induction of apoptosis.

**Fig. 8:**
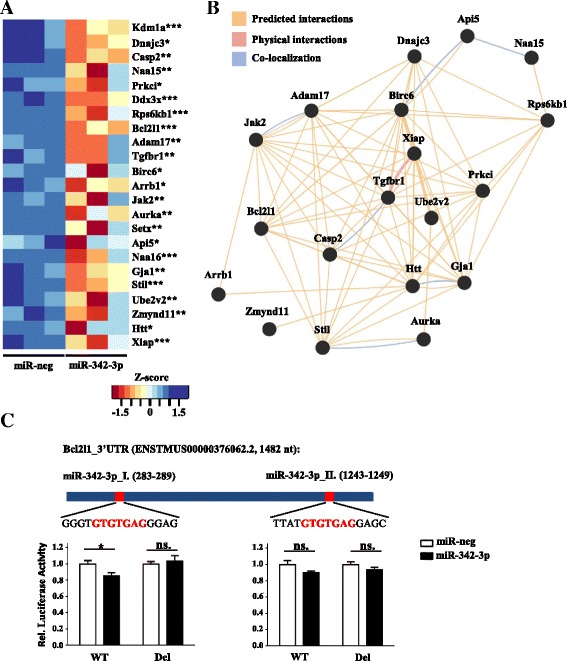
miR-342-3p-repressed anti-apoptotic gene network in macrophages. **a** Relative expression heatmap of selected potential anti-apoptotic miR-342-3p target gene expression in miR-negative control (*miR-neg*) and miR-342-3p-overexpressing RAW264.7 cells 18 h after miRNA mimic transfection in three independent experiments (significance of repression is indicated after the gene name: *FDR ≤0.1, **FDR ≤0.05, ***FDR ≤0.01). **b** GeneMANIA-based identification of the miR-342-3p-repressed, anti-apoptotic gene network. **c** Luciferase activity in HEK293T cells cotransfected with luciferase expression constructs containing WT or mutated (*Del*) miR-342-3p binding sites of Bcl2l1 and miR-342-3p/miRNA negative-control mimics (n = 6). *P ˂ 0.05, *ns.* not significant

Interestingly, the gene set of downregulated potential miR-342-3p target genes contained 23 genes from the GO category “negative regulators of apoptosis” (GO:0043066; Fig. 8a). We used GeneMANIA to investigate the potential interactions between the miR-342-3p-repressed anti-apoptotic genes and found that 19 out of 23 predicted miR-342-3p target genes formed an anti-apoptotic gene network showing extensive predicted interactions and colocalization [73] (Fig. 8b). To explore the direct miRNA–mRNA interactions, we selected Bcl2l1 as one of the central components of the miR-342-3p-regulated anti-apoptotic gene network for further analysis. In silico analysis using the TargetScan target prediction algorithm showed two predicted mir-342-3p binding sites within the 3′ UTR of Bcl2l1 (Fig. 8c, miR-342-3p_I and miR-342-3p_II) [74]. We generated luciferase expression constructs with the predicted miR-342-3p-containing 3′ UTR regions of Bcl2l1 or mutated versions (Fig. 8c). The mutated constructs contained a nine-nucleotide deletion of miR-342-3p binding regions. We cotransfected the generated luciferase expression constructs with miR-342-3p and miRNA-negative control mimics into HEK293T cells. Small but statistically significant repression was observed in the luciferase activity of the miR-342-3p_I binding site-containing construct in the presence of the miR-342-3p mimic compared with the miR-negative control (Fig. 8c). miR-342-3p mimic-dependent reduction of luciferase activity was completely abolished when the miR-342-3p_I binding site was deleted (Fig. 8c). In contrast, the luciferase activity of the construct containing the miR-342-3p_II binding site was not affected significantly by miR-342-3p overexpression. To further examine the specificity of the luciferase assay system we used, we performed two control experiments. First, we detected no activity of the miR-342-3p mimic on the 3′ UTR of Ago2, which does not contain a miR-342-3p binding site. In addition, we did not detect any activity of miR-184 and miR-375 on the 3′ UTR of Bcl2l1-1, which lacks recognition sequences for these control miRNAs (Additional file 12). Taken together, the combination of our in silico and experimental approaches suggest the miR-342-3p-dependent repression of Bcl2l1 expression, however, the demonstration of direct regulation requires further analysis.

## Discussion

Many Th1- and Th2-type inflammation-linked miRNAs, including miR-155, miR-146, and miR-324-5p, appear to be conserved in both mouse and human macrophages and are induced by LPS or IL-4, suggesting that the regulation of miRNA expression and function during classic or alternative macrophage activation is conserved [15, 18, 20, 24, 75].

Our data presented here suggest that additional conserved miRNAs exist in alternatively activated macrophages. We studied the regulation of miR-342-3p, miR-99b, and miR-125a-5p expression during IL-4-induced differentiation in vitro in human and murine macrophages as well as in nematode implantation-induced alternatively activated murine macrophages in vivo. We found that macrophage-specific, IL-4-dependent regulation of these miRNAs showed conserved regulation in human and mouse. It has been previously shown that these miRNAs are expressed in myeloid cells and participate in the regulation of macrophage phenotype, but their function in alternative macrophage activation has not been described before [24, 76–80].

miR-125a and miR-125b, containing the same seed sequences, are induced during classical macrophage activation and potentiate M1 polarization through direct inhibition of anti-inflammatory Tnfaip3 (A20) and Irf4 expression [24, 81, 82]. Intriguingly, we found that miR-125a expression is reduced during alternative macrophage activation both in vitro and in vivo. It has been previously described that the common miR-125a/miR-125b target gene Irf4 facilitates M2 macrophage polarization and host response against helminth infection [83, 84]. In addition, several target prediction algorithms predict Irf4 as a direct target gene of miR-99b. These data raise the possibility that decreased miR-125a and miR-99b levels might contribute to alternative macrophage activation by leading to increased Irf4 expression. However, miR-125b shows opposite regulation during in vitro and in vivo alternative macrophage activation, though regulation of miR-125b expression was not studied at early time points of nematode infection-induced M2 macrophage activation [19]. These findings collectively suggest a complex role of miR99b, miR-125a, and miR-125b in the regulation of alternative macrophage activation. Nevertheless, further studies are needed to explore the time-dependent regulation and dissect the exact roles of these miRNAs during M2 polarization.

IL-4 stimulation leads to the activation of both the JAK/STAT6 and PI3K signaling pathways in myeloid cells [6]. However, it was previously unknown which pathways play critical roles in the regulation of the macrophage microRNome during IL-4-dependent alternative macrophage activation. We observed that the majority of IL-4-regulated miRNAs were strictly STAT6-dependent in mouse macrophages, including miR-342-3p, miR-125a-5p, and miR-99b-5p, as well as the previously studied miR-511-5p and miR-324-5p. In addition, genome-wide mapping of Stat6-binding in IL-4-stimulated T cells showed that activated Stat6 binds intronic and exonic regions as well as distant enhancer regions of its target genes [71, 85]. Accordingly, we found that IL-4 induces Stat6 binding of an adjacent genomic region of Evl in mouse and human macrophages (+4 kb and −4 kb in mouse; +0.3 kb in human), suggesting that Evl and miR-342 are direct targets of IL-4/Stat6 signaling in both human and murine alternative macrophage activation. Interestingly, elevated expression of miR-342-5p (derived from the 5′ strand of the pre-miR-342 miRNA precursor) in inflammatory macrophages has been linked to the formation of atherosclerotic lesions [86]. Both these results and our findings suggest that pri-miRNA expression and miRNA strand selection may be regulated in macrophages by different external stimuli. Interestingly, 11 miRNAs showed IL-4-dependent regulation in STAT6 deficient macrophages, suggesting the participation of STAT6-independent signaling pathway(s) in the regulation of these miRNAs.

Rapid macrophage accumulation is observed in different pathological conditions, including pathogen infections and inflammation [2]. Although the general paradigm of macrophage accumulation is that monocytes or other hematopoietic cells are recruited to sites of inflammation and differentiate into macrophages [87, 88], the rapid proliferation of resident macrophages in adipose tissue of obese animals and atherosclerotic lesions was also described recently [89, 90]. In addition, local IL-4-dependent macrophage proliferation was observed following parasite infections in mice [27]. Certain miRNAs responding to external stimuli have anti-proliferative or pro-apoptotic effects in immune or cancer cells, which may function as a negative feedback mechanism controlling local cell proliferation. Specifically, E2F1 induces cell cycle progression but also potentiates apoptosis via upregulating pro-apoptotic miR-449a/b expression [91]. Furthermore, Th2-type inflammation induces macrophage proliferation and anti-proliferative miR-378 expression simultaneously, suggesting a complex regulation of in situ macrophage proliferation [19]. miR-342-3p also has pro-apoptotic and anti-proliferative roles in colorectal and breast cancer cells [58, 59, 63]. We found that increased miR-342-3p expression paralleled macrophage proliferation changes observed at the early stage of *B. malayi*-induced alternative macrophage activation, which suggests a potential role of miR-342-3p in regulating the amount of local viable macrophages [19]. Indeed, our functional studies showed that miR-342-3p over-expression reduced viable macrophage number via induction of apoptosis. We identified a miR-342-3p-repressed anti-apoptotic gene network with well characterized inhibitors of apoptosis, including Bcl2l1, Xiap, Api5, and Birc6 in macrophages by applying a combination of in silico target prediction algorithms and miRNA mimic experiments. In addition, we found that Bcl2l1 is directly repressed by miR-342-3p. These findings strongly suggest a role for IL-4-induced miR-342-3p as a potent negative feedback regulator of macrophage cell number via induction of apoptosis. Thus, the IL-4-triggered proliferative response of macrophages might be accompanied by the simultaneous induction of counteracting cellular processes, generating an endogenous limit of macrophage abundance. The proposed dual role of IL-4 is further supported by the observation that IL-4 is capable of sensitizing macrophages to rapamycin-induced apoptosis [92]. Intriguingly, elevated expression of miR-342-3p in the liver is accompanied by enhanced macrophage apoptosis in malaria-infected mice [93, 94]. These results thus raise the possibility of a connection between macrophage apoptosis and elevated miR-342-3p expression induced by Th2-type inflammation.

The functional plasticity of macrophages determined by polarization signals plays an important role during the development and progression of several human diseases, including sepsis, malignancies, and metabolic disorders [95]. Alternatively activated macrophage-like disease-associated macrophages are observed in the late phase of sepsis and during tumor progression [96, 97]. In addition, an increased number of alternatively activated-like tumor-associated macrophages has been shown to be associated with poor prognosis in several human tumors, including prostate, cervix, breast, and bladder cancer [98]. For these reasons, the transcriptomic and functional characterization of disease-associated macrophages certainly has a high clinical importance. Based on the data presented here and published by others, macrophage-expressed miRNAs show polarization-specific expression patterns and functions [14–17]. The molecular stability of miRNAs in formalin-fixed, paraffin-embedded samples makes them suitable diagnostic and prognostic markers. Furthermore, a well characterized alternative activation-specific marker set has not been available before in the case of human macrophages [6]. Some miRNAs from our study, including miR-342-3p and miR-193b, may be promising candidates as potential biomarkers in combination with other well characterized alternative macrophage activation-specific genes and proteins in human diseases. Recent studies have provided strong evidence that miRNA targeting in vivo using chemically modified oligonucleotides can alter disease outcome in animal models. It would be interesting to examine if in vivo overexpression of pro-apoptotic miR-342-3p has therapeutic relevance in alternative macrophage activation-associated human diseases, including fibrosis and certain malignant tumors that are characterized by pathologic macrophage abundance. However, both diagnostic and therapeutic applications of these miRNAs require further investigation by utilizing disease models and extensive analyses of clinical samples.

## Conclusions

The aim of this study was to identify the miRNA signature and its phenotypic consequences in human and mouse models of alternative macrophage activation in vitro and in vivo using a combination of transcriptomic, genomic, bioinformatic, and functional approaches. We uncovered a dynamically regulated miRNA expression pattern during human monocyte–macrophage differentiation and IL-4-mediated alternative macrophage activation. We found that three IL-4-responsive miRNAs (miR-342-3p, miR-125a, and miR-99b) showed conserved regulation in both human and mouse alternatively activated macrophages in vitro and in *B. malayi* nematode-infected mice in vivo. We also showed that the alternative macrophage activation-specific regulation of these miRNAs is IL-4Rα- and STAT6-dependent. In addition, we determined that both miR-342-3p and its host gene, EVL, are co-regulated directly by STAT6 in mouse and human macrophages. Finally, we demonstrated that macrophage survival was reduced via miR-342-3p-dependent repression of an anti-apoptotic gene network. These results are consistent with the notion that IL-4/STAT6 signaling-induced miR-342-3p is a potent negative feedback regulator of macrophage cell number via induction of apoptosis.

## Abbreviations

AV, annexin V; BMDM, bone marrow-derived macrophage; ChIP, chromatin immunopreciptiation; FDR, false discovery rate; GO, Gene Ontology; GRO-seq, global run-on sequencing; IL, interleukin; IL-4Rα, IL-4 receptor-α; JAK, Janus kinase; KO, knockout; LPS, lipopolysaccharide; MCSF, macrophage colony stimulating factor; miRNA, microRNA; mRNA, messenger RNA; PI, propidium iodide; PI3K, phosphoinositide 3-kinase; qPCR, quantitative PCR; RMA, robust multi-array average; RT, reverse transcription; STAT1, signal transducer and activator of transcription; TSS, active transcription start site; UTR, untranslated region; WT, wild type.

## Additional files

Additional file 1: List of qPCR assays and primer sequences for gene expression analysis. (XLSX 11 kb) Additional file 2: List of primer sequences for ChIP-qPCR analysis and 3'UTR cloning. (XLSX 10 kb) Additional file 3: Schematic representation of the applied human and mouse alternative macrophage activation protocols. (PDF 345 kb) Additional file 4: Verification of human and mouse alternative macrophage activation based on measurement of alternative macrophage activation markers. (PDF 283 kb) Additional file 5: List of human peripheral blood-derived monocytes and differentiating macrophage-expressed miRNAs. (XLSX 58 kb) Additional file 6: miR-342-3p, miR-193b, miR-99b, miR-125a-5p, and EVL expression in human donor-derived monocytes and macrophages. (PDF 201 kb) Additional file 7: List of IL-4-regulated miRNAs in WT and STAT6 KO macrophages. (XLSX 25 kb) Additional file 8: IL-4-mediated but STAT6-independent regulation of miRNAs in WT and STAT6 KO mouse BMDMs and miR-342-3p-mediated action on IL-4-mediated alternative macrophage activation of RAW264.7 cells. (PDF 20 kb) Additional file 9: Schematic representation of genomic localization of miR-99b and miR-125a coding regions and pri-miR-99b-125a as well as common miR-125a/Spaca6-specific primer pairs. (PDF 7 kb) Additional file 10: List of miR-342-3p mimic-regulated genes in RAW264.7 mouse macrophages. (XLSX 472 kb) Additional file 11: List of predicted mir-342-3p target genes with prediction scores. (XLSX 5370 kb) Additional file 12: Luciferase activity in HEK293T cells cotransfected with luciferase expression constructs containing the miR-342-3p binding site of Bcl2l1 or Ago2 3′ UTRs and miR-342-3p/miR-184/miR-345/miRNA-negative control mimics. (PDF 125 kb)
