# Supplementary material for: The IL-4/STAT6 signaling axis establishes a conserved microRNA signature in human and mouse macrophages regulating cell survival via miR-342-3p
Source: Genome Med. 2016 May 31;8:63. doi: 10.1186/s13073-016-0315-y (PMC4886428; doi:10.1186/s13073-016-0315-y)
Supplement: Additional file 3: — Schematic representation of the applied human and mouse alternative macrophage activation protocols. (PDF 345 kb) [file 13073_2016_315_MOESM3_ESM.pdf]

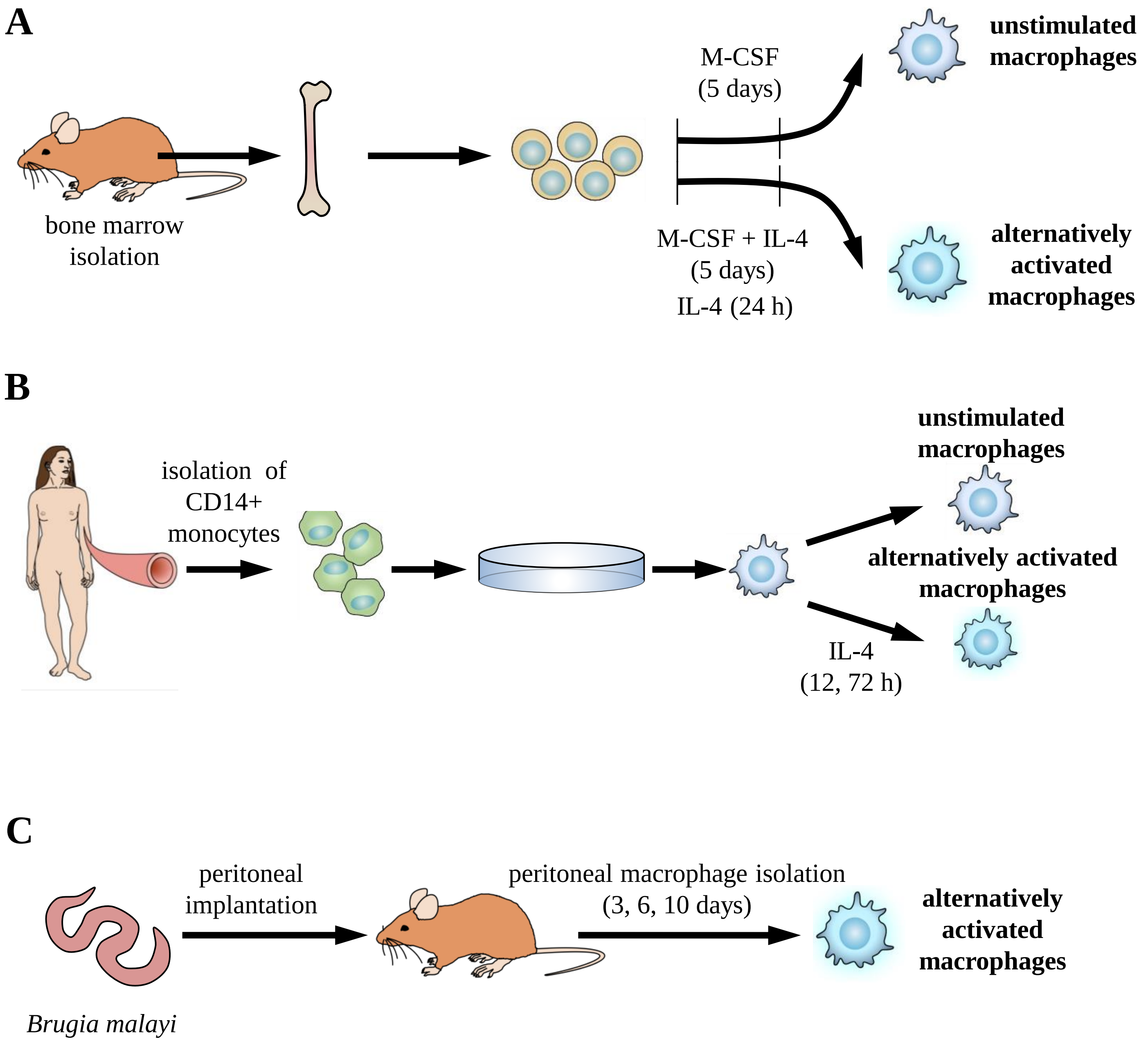

Schematic representation of the applied human and mouse alternative macrophage activation protocols. (A) Schematic representation of human CD14+ monocyte isolation from peripheral blood and the experimental conditions of macrophage differentiation as well as alternative macrophage activation. **(**B) Schematic representation of mouse bone marrow isolation and bone marrow-derived macrophage differentiation in the absence or presence of IL-4. (C) Schematic representation of parasite implantation-induced *in vivo* alternative macrophage activation in mice.
