# Supplementary figures and images for: The IL-4/STAT6 signaling axis establishes a conserved microRNA signature in human and mouse macrophages regulating cell survival via miR-342-3p
Source: Genome Med. 2016 May 31;8:63. doi: 10.1186/s13073-016-0315-y (PMC4886428; doi:10.1186/s13073-016-0315-y)

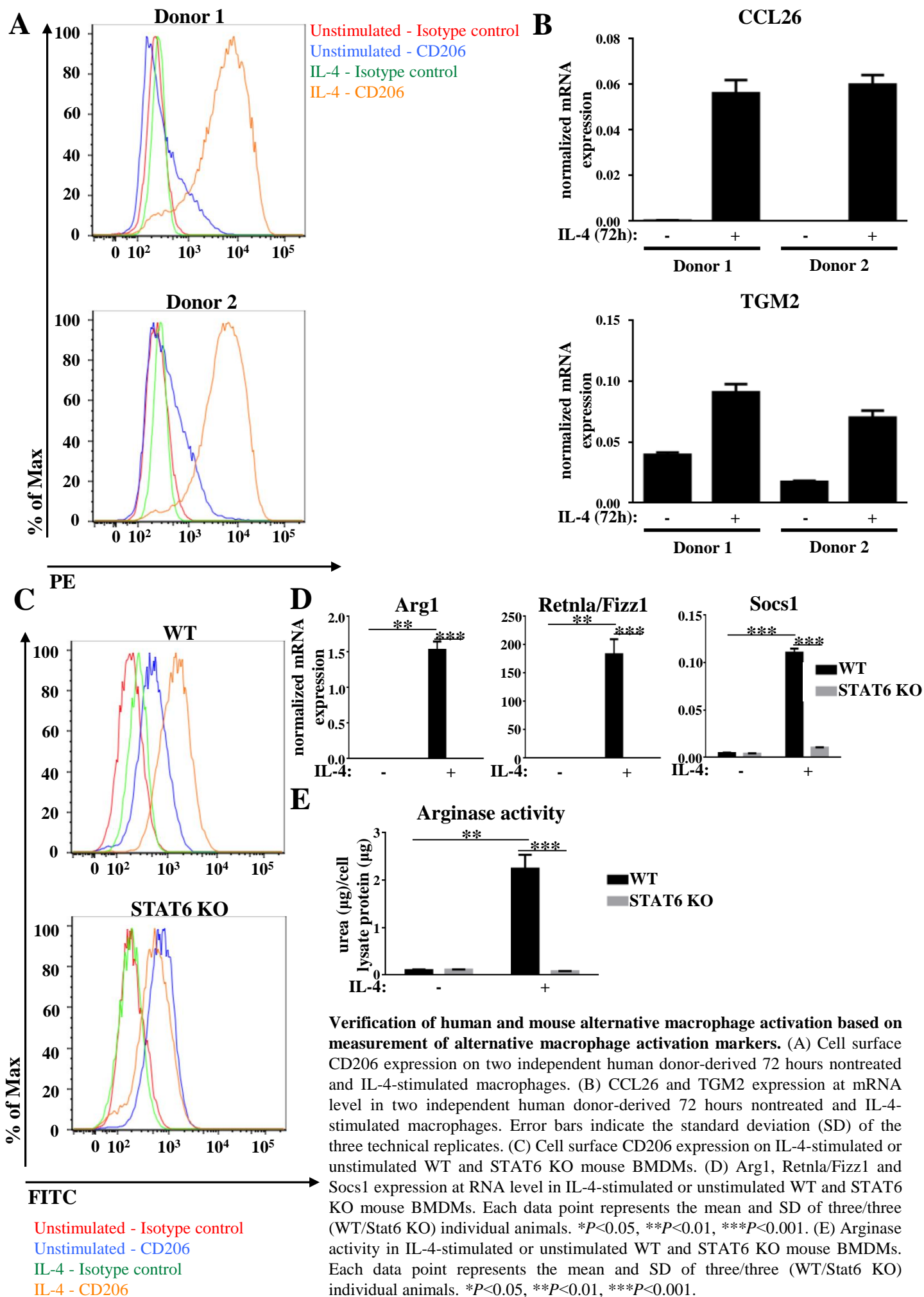

Supplement: Additional file 4: — Verification of human and mouse alternative macrophage activation based on measurement of alternative macrophage activation markers. (PDF 283 kb) [file 13073_2016_315_MOESM4_ESM.pdf]
