# Supplementary material for: The IL-4/STAT6 signaling axis establishes a conserved microRNA signature in human and mouse macrophages regulating cell survival via miR-342-3p
Source: Genome Med. 2016 May 31;8:63. doi: 10.1186/s13073-016-0315-y (PMC4886428; doi:10.1186/s13073-016-0315-y)
Supplement: Additional file 6: — miR-342-3p, miR-193b, miR-99b, miR-125a-5p, and EVL expression in human donor-derived monocytes and macrophages. (PDF 201 kb) [file 13073_2016_315_MOESM6_ESM.pdf]

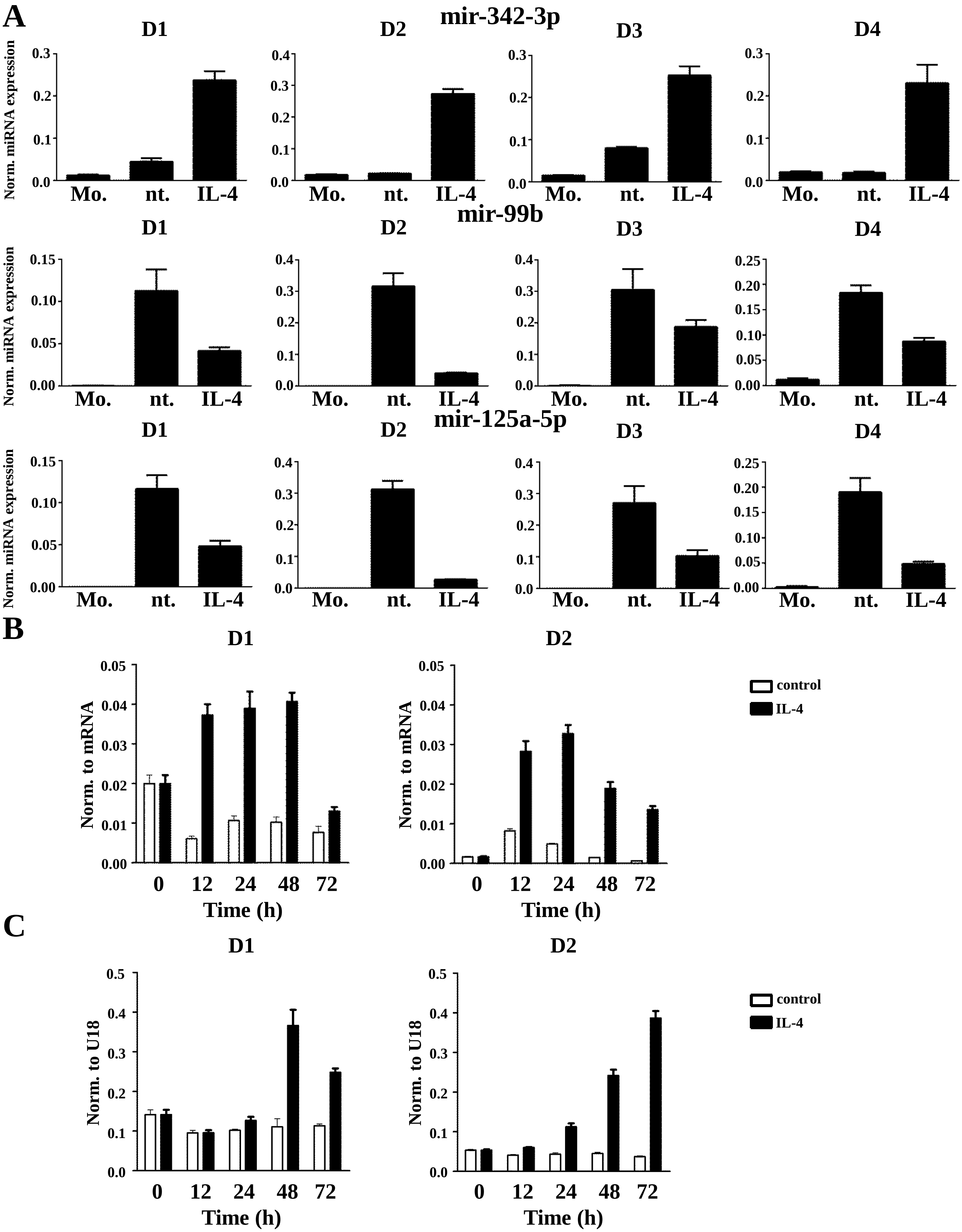


**Mir-342-3p, miR-193b, miR-99b, miR-125a-5p and EVL expression in human donors-derived monocytes and macrophages**. (A) MiR-342-3p, miR-193b, miR-99b and miR-125a-5p expression in four (D1-D4) independent human donor-derived monocytes, 72 hours nontreated and IL-4 stimulated macrophages. Error bars indicate the standard deviation (SD) of the three technical replicates. (B) EVL expression in two other (D2, D3) human donor-derived differentiating macrophages in the absence or presence of IL-4. Error bars indicate the standard deviation (SD) of the three technical replicates. (C) MiR-342-3p in two other (D2, D3) human donor-derived differentiating macrophages in the absence or presence of IL-4. Error bars indicate the standard deviation (SD) of the three technical replicates.
