# Supplementary material for: The IL-4/STAT6 signaling axis establishes a conserved microRNA signature in human and mouse macrophages regulating cell survival via miR-342-3p
Source: Genome Med. 2016 May 31;8:63. doi: 10.1186/s13073-016-0315-y (PMC4886428; doi:10.1186/s13073-016-0315-y)
Supplement: Additional file 8: — IL-4-mediated but STAT6-independent regulation of miRNAs in WT and STAT6 KO mouse BMDMs and miR-342-3p-mediated action on IL-4-mediated alternative macrophage activation of RAW264.7 cells. (PDF 20 kb) [file 13073_2016_315_MOESM8_ESM.pdf]

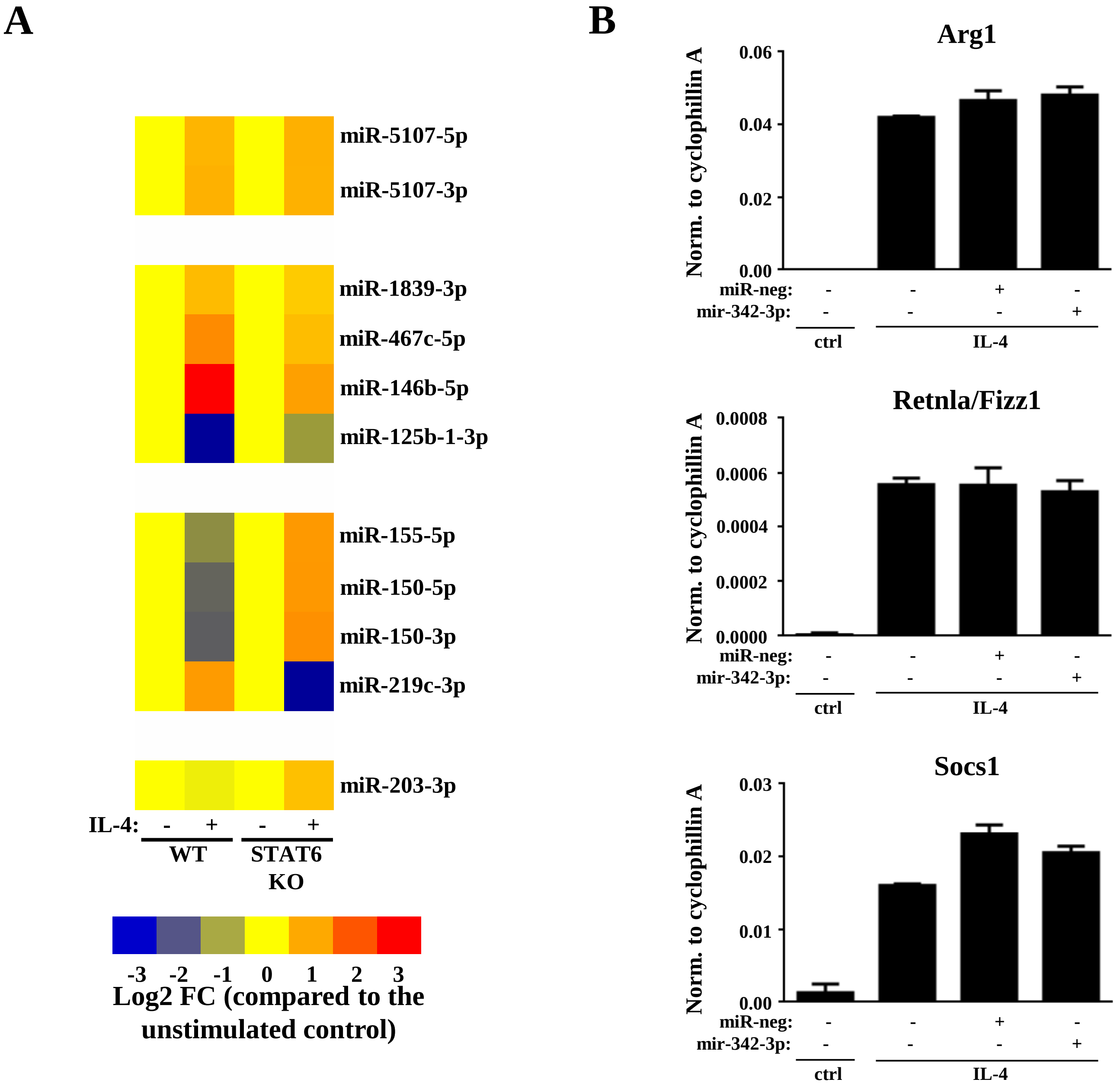

IL-4-mediated but STAT6-independent regulation of miRNAs in WT and STAT6 KO mouse BMDMs and miR-342-3p-mediated action on IL-4-mediated alternative macrophage activation of RAW264.7 cells. (A) Heatmap showing average fold changes of IL-4-regulated but STAT6 independent miRNAs in IL-4-stimulated and untreated mouse BMDMs. Colour intensities reflect the fold changes compared to IL-4-stimulated WT to unstimulated WT as well as IL-4-treated STAT6 KO to untreated STAT6 KO macrophages. (B) Alternative macrophage activation marker Arg1, Retnla/Fizz1 and Socs1 expression at RNA level in mir negative control (miR-neg) and miR-342-3p (miR-342-3p) mimics-transfected RAW264.7 cells in presence and absence of IL-4. A representative of two independent experiments is shown. Error bars indicate the standard deviation (SD) of the three technical replicates.
