# Supplementary material for: The IL-4/STAT6 signaling axis establishes a conserved microRNA signature in human and mouse macrophages regulating cell survival via miR-342-3p
Source: Genome Med. 2016 May 31;8:63. doi: 10.1186/s13073-016-0315-y (PMC4886428; doi:10.1186/s13073-016-0315-y)
Supplement: Additional file 9: — Schematic representation of genomic localization of miR-99b and miR-125a coding regions and pri-miR-99b-125a as well as common miR-125a/Spaca6-specific primer pairs. (PDF 7 kb) [file 13073_2016_315_MOESM9_ESM.pdf]

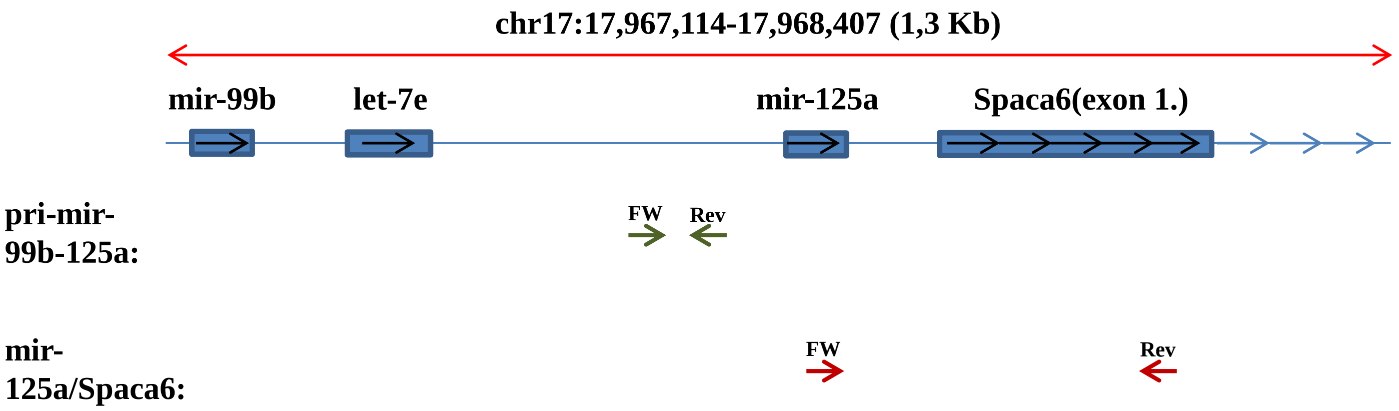

Schematic representation of genomic localization of miR-99b and miR-125a coding regions and pri-miR-99b-125a as well as common miR-125a/Spaca6-specific primer pairs.
