# Supplementary material for: The IL-4/STAT6 signaling axis establishes a conserved microRNA signature in human and mouse macrophages regulating cell survival via miR-342-3p
Source: Genome Med. 2016 May 31;8:63. doi: 10.1186/s13073-016-0315-y (PMC4886428; doi:10.1186/s13073-016-0315-y)
Supplement: Additional file 12: — Luciferase activity in HEK293T cells cotransfected with luciferase expression constructs containing the miR-342-3p binding site of Bcl2l1 or Ago2 3′ UTRs and miR-342-3p/miR-184/miR-345/miRNA-negative control mimics. (PDF 125 kb) [file 13073_2016_315_MOESM12_ESM.pdf]

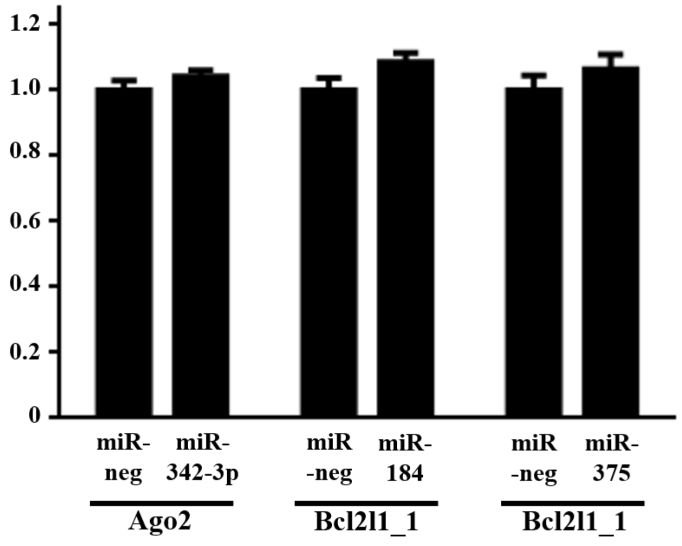

Luciferase activity in HEK293T cells cotransfected with luciferase expression constructs containing miR-342-3p binding site of Bcl2l1 or Ago2 3’UTRs and miR-342-3p/miR-184/miR-345/miRNA negative-control mimics (n=3).
